# A Parasite in Disguise: Hydatid Cyst of the Breast

**DOI:** 10.1590/0037-8682-0237-2026

**Published:** 2026-10-16

**Authors:** Ahmet Tuğrul Akkuş, Fadime Güven

**Affiliations:** 1 Kağızman State Hospital, Kars, Turkey.; 2 Atatürk Üniversitesi Tıp Fakültesi Radyoloji Anabilim Dalı, Erzurum, Turkey.

Hydatid disease (HD) is caused by *Echinococcus granulosus* (hydatid cysts) and *E. multilocularis* (invasive alveolar disease that mimics malignancy)^1^. It is endemic in South America, the Mediterranean, the Middle East, and Australia^1^. The liver is the most common site, followed by the lung and peritoneum^1^. Breast involvement is exceedingly rare and typically occurs in disseminated disease^2^. Imaging is critical for diagnosis because a Tru-Cut® biopsy can result in rupture, dissemination, and anaphylaxis.

A 30-year-old woman presented with a gradually enlarging, palpable breast lump. Ultrasound revealed a well-defined, hyper-isoechoic lesion with no internal vascularity on power Doppler imaging. Figure 1 shows linear serpentine hypoechoic lines representing detached membranes (white arrowhead) and anechoic foci representing daughter cysts (white arrow), findings that are highly suspicious for a hydatid cyst. Enzyme-linked immunosorbent assay (ELISA) was positive for *Echinococcus* IgG. Magnetic resonance imaging (MRI) was performed for further characterization: Figure 2 shows a T2-weighted hyperintense cystic lesion with clearly visible detached internal membranes (black arrow), characteristic of HD. The patient underwent total surgical excision and histopathology confirmed HD.

**FIGURE 1:**
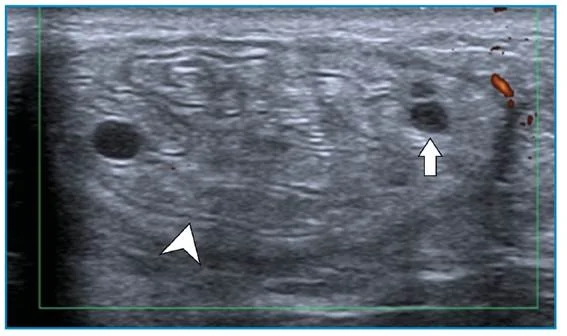
Ultrasound showing a well-defined hyper-isoechoic lesion with no internal flow on power Doppler imaging with linear serpentine hypoechoic lines representing detached membranes (white arrowhead) and anechoic cystic structures representing daughter cysts (white arrow).

**FIGURE 2:**
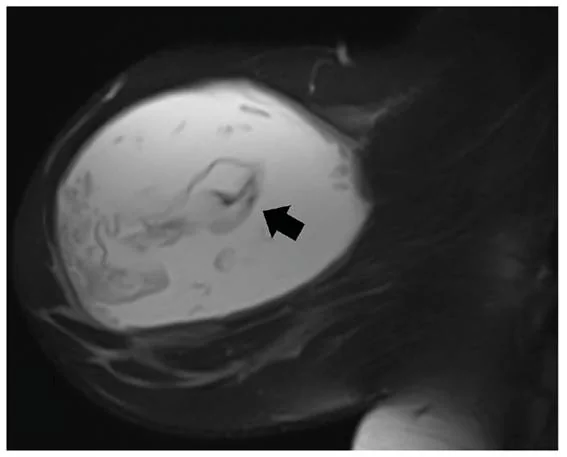
T2-weighted magnetic resonance image showing a hyperintense cystic lesion with detached membranes (black arrow).

Breast HD typically presents as a painless, slowly growing lump^3^. Mammography shows a well-circumscribed mass with possible calcifications, which may mimic a fibroadenoma or phyllodes tumor^4^. Ultrasound appearance varies by cyst stage. MRI is the most specific, with T2-hypointense serpentine membranes pathognomonic for HD^5^. ELISA is less sensitive in extra-hepatic locations^6^. Surgeons must recognize this entity to prevent intra-operative rupture and dissemination.
